# *In vivo*-expression profile and regulation of the antiviral restriction factor CD317/tetherin in humans

**DOI:** 10.1186/1742-4690-8-S2-O9

**Published:** 2011-10-03

**Authors:** Elina Erikson, Tarek Adam, Sarah Schmidt, Judith Lehmann-Koch, Benjamin Over, Christine Goffinet, Christoph Harter, Isabelle Bekeredjian-Ding, Serkan Sertel, Felix Lasitschka, Oliver T Keppler

**Affiliations:** 1 Department of Infectious Diseases, University of Heidelberg, 69120 Heidelberg, Germany; 2 Institute of Pathology, University of Heidelberg, 69120 Heidelberg, Germany; 3 Department of Internal Medicine V, University of Heidelberg, 69120 Heidelberg, Germany; 4 Department of Otolaryngology, Head and Neck Surgery, University of Heidelberg, 69120 Heidelberg, Germany

## Background

Human CD317 (BST-2/HM1.24/tetherin) restricts the release of multiple viruses including HIV-1, XMRV, Lassa virus and KSHV from infected cells in culture. Its relevance for infection control in humans is however unclear, in part due to its poorly defined *in vivo*-expression pattern.

## Materials and methods

To provide a framework for studies into the biological functions, regulation and therapeutic potential of CD317, we performed a tissue microarray-based expression profiling in 468 samples of 25 healthy organs from over 250 patients, not suffering from clinically apparent infections.

## Results

We report that CD317 protein was expressed to varying degrees in all organs tested and detected in a number of specialized cell types including hepatocytes, pneumocytes in the lung, ducts of major salivary glands, pancreas and kidney, Paneth cells in the small intestine, epithelia of multiple organs, Leydig cells in the testis, plasma cells, bone marrow stromal cells, monocytes, and vascular endothelium. Remarkably, many of these CD317-positive cell types are *in vivo*-targets for pathogenic viruses, only for some of which restriction by CD317 or virus-encoded antagonists have thus far been investigated. Of note, major HIV target cells in tonsil and gut-associated lymphoid tissue did not express CD317. Limited, cell type-dependent co-expression of CD317 with the interferon biomarker MxA *in vivo* and an unresponsiveness to cytokine stimulation in lymphoid organ explants suggest that type I interferons may only in part regulate CD317.

## Conclusions

This *in vivo*-expression profiling sheds light on the biology and species-specificity of CD317, identifies multiple novel interaction sites of viruses with this restriction factor, and refutes the widely-held belief of its restricted constitutive expression and primary interferon inducibility. Work is in progress to define anatomical compartments and/or pathological conditions under which CD317 is expressed in HIV-1 target cells *ex vivo* and *in vivo.*
